# Uterine Artery Embolization Versus Hysterectomy in the Treatment of Symptomatic Adenomyosis: Protocol for the Randomized QUESTA Trial

**DOI:** 10.2196/resprot.8512

**Published:** 2018-03-01

**Authors:** Annefleur Machteld de Bruijn, Paul NM Lohle, Judith AF Huirne, Jolanda de Vries, Moniek Twisk, QUESTA-Trial Group, Wouter JK Hehenkamp

**Affiliations:** ^1^ Department of Gynaecology and Obstetrics Vrije Universiteit Medical Center Vrije Universiteit Amsterdam Netherlands; ^2^ Amsterdam Cardiovascular Sciences Vrije Universiteit Amsterdam Netherlands; ^3^ Department of Radiology Elisabeth-TweeSteden Ziekenhuis Tilburg Netherlands; ^4^ Department of Medical and Clinical Psychology Tilburg University Tilburg Netherlands; ^5^ Department of Medical and Clinical Psychology Elisabeth-TweeSteden Ziekenhuis Tilburg Netherlands; ^6^ Department of Gynecology Medical Center Zuiderzee Lelystad Netherlands

**Keywords:** adenomyosis, uterine artery embolization, hysterectomy, randomized trial, quality of life

## Abstract

**Background:**

Adenomyosis is a benign uterine disease characterized by invasion of endometrium into the myometrium resulting in heavy menstrual bleeding and pain (dysmenorrhea). Hysterectomy is established as the final treatment option when conservative treatment fails. Uterine artery embolization (UAE) in patients with symptomatic adenomyosis has demonstrated to reduce symptoms and improve quality of life. However, randomized controlled trials are lacking.

**Objective:**

With this study, we aim to evaluate the impact of UAE on Health-Related Quality of Life (HRQOL) in a randomized comparison to hysterectomy in patients with symptomatic adenomyosis.

**Methods:**

This is a multicenter non-blinded randomized controlled trial comparing UAE and hysterectomy. Eligible patients are symptomatic premenopausal women without the desire to conceive and who have symptomatic magnetic resonance imaging (MRI)–confirmed pure adenomyosis or dominant adenomyosis accompanied by fibroids. After obtaining informed consent, patients will be randomly allocated to treatment in a 2:1 UAE versus hysterectomy ratio. The primary objective is HRQOL at 6 months following the assigned intervention. Secondary outcomes are technical results, pain management, clinical outcomes, HRQOL, and cost effectiveness during 2 years of follow-up. In addition, transvaginal ultrasound (TVUS) and MRI will be performed at regular intervals after UAE.

**Results:**

Patient enrollment started November 2015. The follow-up period will be completed two years after inclusion of the last patient. At the time of submission of this article, data cleaning and analyses have not yet started.

**Conclusions:**

This trial will provide insight for caretakers and future patients about the effect of UAE compared to the gold standard hysterectomy in the treatment of symptomatic adenomyosis and is therefore expected to improve patients’ wellbeing and quality of life.

**Trial Registration:**

Netherlands Trial Register NTR5615; http://www.trialregister.nl/trialreg/admin/rctview.asp?TC=5615 (Archived by WebCite at http://www.webcitation.org/6xZRyXeIF)

## Introduction

Adenomyosis is described as the benign presence of ectopic endometrial glands and stroma causing reactive hypertrophy of the smooth muscle fibers of the myometrium [1,2]. The prevalence of adenomyosis is estimated to be 5%-8% in some studies, whereas others find even 40%-70% [3-5]. Approximately one-third of women with adenomyosis are symptomatic [2]. Symptoms associated with the presence of adenomyosis are abnormal menstrual bleeding, pain (dysmenorrhea) and an enlarged uterus. About 40%-50% and 15%-30% of patients will suffer from heavy menstrual bleeding and/or dysmenorrhea, respectively [3]. Fibroids are present in up to 55% of the patients diagnosed with adenomyosis [6]. Therefore, it can be difficult attributing symptoms to one or the other [7,8]. Adenomyosis can be diagnosed with transvaginal ultrasonography (TVUS) or magnetic resonance imaging (MRI) [9,10]. Adenomyosis can be treated conservatively (hormonal/non-hormonal). When conservative management fails, a hysterectomy is the most common surgical solution, since surgical removal of adenomyosis is difficult given its diffuse aspect.

Uterine artery embolization (UAE) has been a minimally invasive treatment for symptomatic uterine fibroids since 1995 [11]. Since then, much research has been conducted including several randomized controlled trials establishing UAE as a valuable treatment option for women with symptomatic fibroids [12-14].

During the last fifteen years, several case series and cohorts evaluated UAE as a treatment for patients suffering from symptomatic adenomyosis. These cohorts show promising results [15-26]. Randomized data, comparing this new treatment modality with the gold standard (ie, hysterectomy) are lacking though. The “Quality of Life after Embolization vs Hysterectomy in Adenomyosis” (QUESTA) trial was set up to fill this knowledge gap comparing UAE with hysterectomy in patients with symptomatic adenomyosis. In this paper, we present the design of the trial.

## Methods

### Design

The QUESTA trial is a multicenter nonblinded randomized controlled trial, performed within selected hospitals in the Netherlands containing experienced interventional radiologists qualified to perform UAE. The study is performed in a network infrastructure in which radiologists and gynecologists collaborate. This trial will be conducted in accordance with the Consolidated Standard of Reporting Trials [27-29], the principles of the Declaration of Helsinki, and the Medical Research Involving Human Subject Act.

This study is approved by the ethics committee of the VU Medical Centre Amsterdam (Reference Number 2015/211) and by the boards of all participating hospitals. The trial is registered at the Netherlands Trial Registry (Netherlands Trial Register NTR5615).

### Participants and eligibility criteria

Eligible adult women are asked to participate when they meet the following inclusion criteria:

1. Premenopausal women with symptomatic pure adenomyosis or dominant adenomyosis (with concurrent uterine fibroids). Symptoms are defined as heavy menstrual bleeding, dysmenorrhea, and/or cycle independent pain and bulk-related symptoms.
2. Women with an indication for hysterectomy. These women had or have unsuccessful medicinal treatment or decided that such treatment is no option.

The exclusion criteria are:

1. Patients younger than 18 years of age
2. Patients with a pelvic infection
3. Suspicion or presence of a malignancy
4. Current pregnancy or desire to conceive in the future
5. Absolute contraindication for angiography
6. Deep infiltrating endometriosis requiring surgery or risks on intestinal stenosis
7. Concurrent hysteroscopic removable submucous fibroids

### Procedures, recruitment, and randomization

When adenomyosis is suspected on TVUS (see criteria in Table 1) and MRI is performed to confirm adenomyosis (see criteria in Table 2), eligible patients will be informed about the study by the gynecologist, resident, research nurse or study coordinator and will be informed about the website for additional information and an introduction video (Multimedia Appendix 1). If the patient declines randomization, she will be asked to participate in the cohort group. The patients in the cohort study group will be offered standard care (ie, hysterectomy, since embolization is not available outside the trial). Afterwards, participants with written informed consent are randomly allocated (2:1) to the experimental intervention (UAE) or the standard care control group (hysterectomy). Randomization is computer-based and stratified for the participating hospitals. The study is not blinded since uterine artery embolization is performed either under conscious sedation or epidural anesthesia in contrast to a hysterectomy which is performed in the operating room under full narcosis. Therefore, it is not possible to blind the patients or the physicians.

### Intervention group

A specific UAE particle protocol for adenomyosis will be delivered to the interventional radiologist of each center and, if needed, our experienced interventional radiologist will be present during the first UAE procedure (PNM Lohle).

**Table 1:** Criteria for diagnosing adenomyosis on transvaginal ultrasonography (TVUS).

Criteria	eCRF^a^ answers
Uterus (cm)	Height*length*width
Asymmetrical thickening^b^	Yes/no/I don’t know; Measurements uterine walls
Cysts^b^	Yes/no/I don’t know
Fan-shaped shadowing^b^	Yes/no/I don’t know
Myometrial aspect^b^	Homogenous/inhomogeneous/I don’t know
Inhomogeneous endometrial-myometrial zone (endometrial lines/buds)^b^	Yes/no/I don’t know
Diffuse flow^b^	Yes/no/I don’t know
Adenomyosis type	Diffuse/Focal/Combined diffuse>focal/Combined focal>diffuse
Fibroids	Yes/no/I don’t know
Fibroid count	Number
Size biggest fibroids (cm)	Height*length*width
Occurrence of pedunculated fibroid	Yes/no
Pedunculated fibroid count	Number

^a^eCRF:electronic clinical report forms.

^a^Adenomyosis criteria. If 3/6 are recognized, adenomyosis is suspected.

**Table 2:** Criteria for diagnosing adenomyosis on magnetic resonance imaging (MRI).

Criteria and types	Definition
Adenomyosis	Junctional zone^a^>12mm diameter
Cysts (hyper intense foci T2)	Yes/no
Asymmetric myometria	Yes/no
Adenomyosis category 1	Focal: 25% or less of endometrial interface
Adenomyosis category 2	Regional: Entire endometrial surface of the anterior wall, posterior wall or fundus
Adenomyosis category 3	Diffuse: Entire or most of the endometrial surface Moderate: 20-29mm Severe: ≥30mm
Dominant adenomyosis in combination with fibroid	Volume domination: Adenomyosis>fibroids

^a^Adenomyosis is confirmed.

UAE is carried out under epidural anesthesia or patient controlled analgesia (PCA). A catheter is introduced in the femoral artery and positioned selectively into the uterine arteries under fluoroscopic guidance. Microspheres are then injected through the catheter into the uterine artery. The bloodstream will move the microspheres towards the small uterine artery branches in the area of adenomyosis (and fibroids if present). Microspheres consist of a hydrogel core with polymethylmethacrylate (PMMA) and a flexible shell of polyphosphazene (Polyzene-F), which is a synthesized inorganic biostable and biocompatible polymer (Embozene microsphere). This embolic agent establishes reduction and cessation of blood flow to the area of adenomyosis resulting in ischemia and infarction. The embolization protocol sets out the provisions regarding the microsphere size (Embozene 500 µm) for use and the angiographic embolization end-point until full stasis at the distal end of the uterine artery.

A second protocol will include: administration of antibiotics, drip infusions, Foley bladder catheters, PCA pump usage with strict protocols for pain management, and a nursing protocol for the ward.

### Control group

Hysterectomy is preferably performed by vaginal hysterectomy (VH) or total laparoscopic hysterectomy (TLH). A TLH or abdominal hysterectomy is always performed under general anesthesia. No protocol will be provided for surgical standardization. Adhesiolysis will be performed when necessary. Planned concomitant endometriosis surgery serves as an exclusion criterion. However, during surgery, coagulation of mild endometriosis is allowed. In case of laparoscopic or vaginal hysterectomy, the uterus will be removed vaginally or by the use of (in bag) morcellation. Supra cervical hysterectomy is allowed.

### Data collection

**Figure 1:**
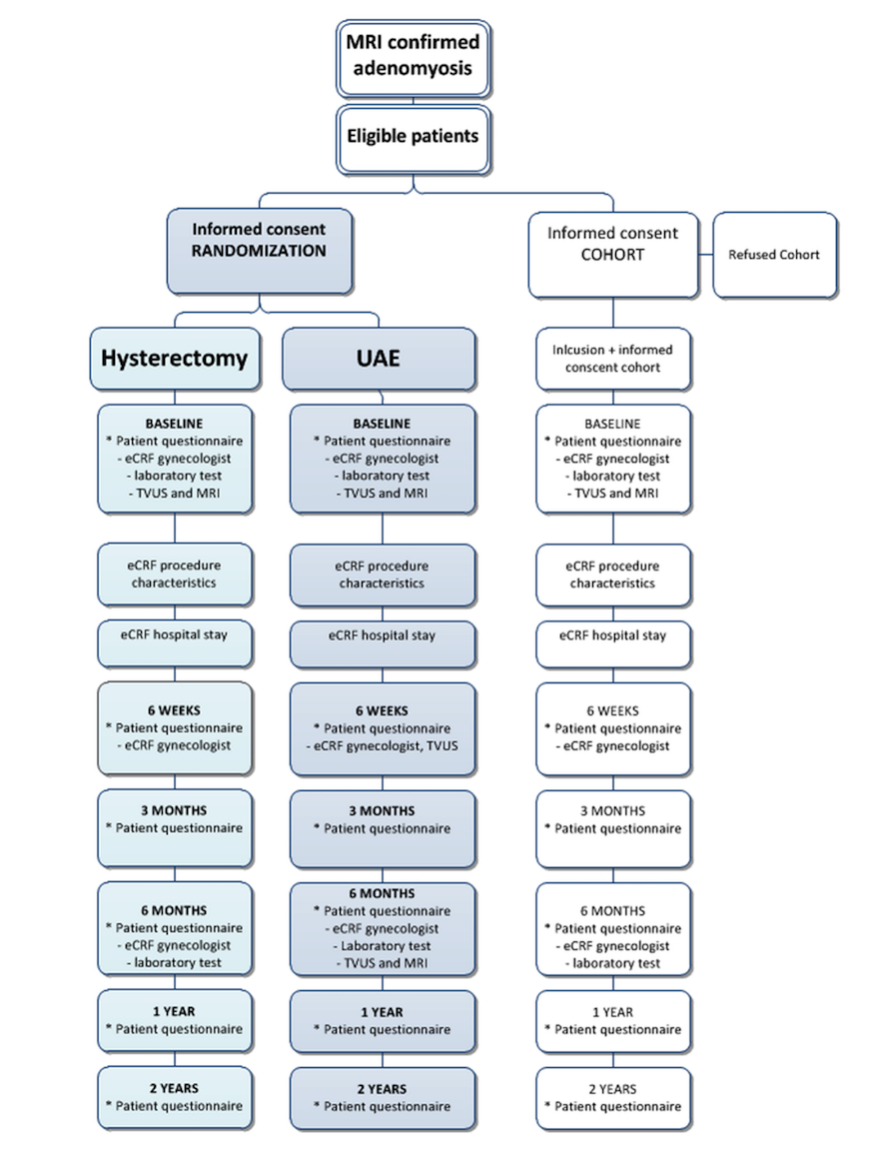
Study flowchart. Patient data includes the following questionnaires: Pictorial Blood Assessment Chart (PBAC), Numeric Pain Rating Scale (NRS), Facet 1:Pain and discomfort WHOQOL-100, World Health Organization Quality Of Life-Bref (WHOQOL-Bref), Short Form-12 (SF-12), Facet 15: sexual activity domain WHOQOL-100, recovery (RI-10), Euro-QOL 5D (EQ-5D), patient preference (vignette), and patient satisfaction (Likert-scale).

All electronic clinical report forms (eCRF) and patient questionnaires are digitally online secured and filled out in the study website with the use of a patient-specific study number [30]. The patient and physician receive this study number at time of informed consent. Figure 1 shows the study flowchart.

### Baseline

At time of inclusion and randomization, baseline medical history, obstetric history, and laboratory work up (hemoglobin, renal function (eGFR), CA-125, Anti-Mullerian hormone (AMH) in a subset of centers) are reported in the eCRF. The imaging characteristic displayed in Table 1 will all be registered.

Patients will fill out validated Health-Related Quality of Life (HRQOL) and symptom questionnaires (see "outcome measures")

### Procedure

At procedure, data about the course of the intervention (UAE or operation), any particularities or complications are reported in the eCRF. At discharge from the hospital the eCRF will report the total of admitted days and complications during hospital stay.

### Follow-up

The research investigator will send invitations for the digital online secured patient questionnaires by email at baseline, 6 weeks, 3 months, 6 months, 12 months and 24 months of follow-up. All patients will also specifically be asked at baseline to give their consent to be approached for long-term follow up. Validated questionnaires will be filled out to report on HRQOL, symptoms, clinical outcomes, return to normal activities, absence of work, medication use, costs, medical consultation/consumption, and additional received therapy (see “outcome measures”). The patients who underwent UAE will receive a TVUS at 6 weeks and 6 months with an MRI at 6 months to compare the adenomyosis features with baseline results. In the hysterectomy group, pathology outcomes are also registered. All the eCRFs will report complications.

### Outcomes measures

The primary outcome is quality of life at 6, 12 and 24 months after index procedure as measured by a combination of the World Health Organization Quality of Life Scale (whoqol-Bref) and Short Form-12 (SF-12) Questionnaire.

Secondary outcomes at 6 weeks and 3, 6, 12, 24 months after treatment consists of:

1. Clinical outcomes: technical failure rate, clinical failure arte as defined by secondary hysterectomy, additional therapy or reinterventions, complications.
2. Recovery related outcomes (6 weeks, 3 and 6 months): hospital stay, return to normal activities (Recovery Index-10).
3. Symptom and quality of life outcomes: menstrual characteristics (pictorial blood assessment chart), validated pain-questionnaire (Numeric Pain Rating Scale 0-100 scale, Facet 1: Pain and discomfort WHOQOL-100), sexual functioning (WHOQOL-100, sexual activity domain), satisfaction (Likert-scale, vignettes preference), quality of life (WHOQOL-Bref, SF-12).
4. Cost utility analysis (European Quality of Life 5 Dimensions) after 12 months.
5. Laboratory outcomes (baseline, 6 weeks and 6 months): hemoglobin, CA-125 and AMH in specific predefined centers.
6. Pathology outcomes (6 weeks): pathological finding of uterus in hysterectomy group.

Also, imaging outcomes will be investigated in order to identify potential predictive parameters for therapy effect.

1. TVUS (baseline, 6 weeks and 6 months): imaging parameters described in Table 1, uterine size reduction and reduction of fibroid volume in case of concomitant fibroids. In specific predefined centers, we will measure vascular index (3D power Doppler).
2. MRI (baseline and 6 months): imaging parameters described in Table 1, uterine size reduction, junctional zone reduction, infarction rate, reduction of fibroid volume in case of concomitant fibroids, presence of endometriosis.

### Sample size

This study has a noninferiority design, where UAE is considered noninferior to hysterectomy when HRQOL at 6 months does not differ (delta) more than 5 points (scale 0-100). When the following assumptions are used: SD of 9 points (scale 0-100), alpha 0.10, power 0.80, using a one-sided two-sample equal-variance *t* test, a sample size of 1 x 52 patients (embolization) and 1 x 34 patients (hysterectomy) is needed. Excluding 10% dropout, in total 96 patients are needed for the primary outcome. Assumptions are based on the Embolization versus Hysterectomy (EMMY) trial outcomes [13].

### Statistical analysis

Analysis of the study will be based on the intention-to-treat and per protocol analysis. With regard to the primary outcome variable (WHOQOL-Bref, SF-12), non-inferiority is established between UAE and hysterectomy when the mean difference does not exceed 5 points (with a SD of 9 points).

The primary outcome will be analyzed using linear mixed modeling, applying transformation if necessary, adjusting for, if necessary, clinically relevant baseline imbalances. With regard to the secondary outcomes, we will use the appropriate nonparametric and parametric statistics to evaluate statistically significant differences between the two treatments. A *P* value <.05 will be considered as statistically significant. In all analyses, statistical uncertainties will be expressed in 90%CI.

The database will be locked 6 months after the last surgical procedure in order to obtain the short-term outcomes. These data will be analyzed and published in a “short term results” manuscript. The long-term results (HRQOL and costs) will be analyzed 12 months after the last procedure, when the last patient returned her last questionnaire.

### Safety Monitoring and Interim Analysis

Since both treatment options have proven their safety, no specific risks apply. This study is conducted to determine the efficacy of the treatment. However, safety monitoring without direct involvement in the trial (clinical research bureau, VUMC) will be installed to monitor the setup and conduct of the trial. No interim analysis is planned because of the relatively small sample size.

All serious adverse events (SAEs) will be reported to the accredited METC that approved the protocol, within 15 days after the sponsor has first knowledge of the serious adverse events. SAEs that result in death or are life threatening should be reported expedited. The expedited reporting will occur no later than 48 days after the responsible investigator has first knowledge of the adverse event. In case of more than one SAE, the METC will be notified. This committee can advise to terminate the trial due to safety reasons.

## Results

Inclusion of patients started in November 2015. The expected end date is November 2019. Data collection for the primary outcome will be expected to last until May 2020. Data collection concerning the secondary outcomes are expected two years following the last included patient. Analysis has not yet started as of this article’s submission.

## Discussion

### Summary

With this randomized controlled trial, we aim to evaluate the impact of UAE on HRQOL in a randomized comparison to hysterectomy in patients with symptomatic adenomyosis.

### Strengths and Limitations

This is the first randomized controlled trial evaluating effectiveness of UAE versus hysterectomy in patients with symptomatic adenomyosis. The QUESTA trial uses a web-based randomization program with the use of allocation concealment which reduces the chance of selection bias. The study is not blinded for the patient or health care worker which could possibly influence the outcomes. Blinding is impossible considering the nature of the treatments. Earlier studies reported on UAE being more cost-effective compared to hysterectomy, however these studies were conducted in patients with fibroids and mostly carried out through abdominal hysterectomies [31]. Over the years hysterectomy techniques have changed and the more cost-effective laparoscopy has become available. No studies have yet reported on cost effectiveness of UAE versus laparoscopic hysterectomy. We expect UAE to be more cost effective since the procedure itself is less expensive and recovery time is shorter [32], however we do expect consultation to be more frequent in the UAE group. We note that we allow all hysterectomy techniques in this study due to the intention to treat analysis. It will be registered and corrected for in analysis.

Disease specific questionnaires for adenomyosis have not yet been developed. Used questionnaires are validated in terms of quality of life, pain, heavy menstrual bleeding, sexual functioning, recovery, and allocation satisfaction and proved to be disease specific in the EMMY trial [13].

The follow-up in this trial is set at 24 months. This could be a limitation since 5- and 10-year follow-up of the EMMY trial showed additional hysterectomies in the UAE group [33,34]. Depending on the result of this trial a possibility to extend follow-up was included in the informed consent.

In the last 15 years, 30 cohorts and case series [15-26,35-52] described UAE in the treatment of symptomatic adenomoysis. The lack of level 1 evidence, heterogeneous particle use and UAE techniques make the development of a national guideline for standardized UAE in the treatment of adenomyosis challenging. We provide a mini-protocol on the usage of the standardized microspheres, however we do not provide a specific UAE technique protocol because we assume general UAE techniques will be followed [28]. In addition, we wish to maintain the intention to treat analysis of the Dutch population treated in the UAE centers.

### Potential Impact and Implications

Results of the QUESTA trial will provide knowledge for the most optimal treatment regimen in terms of HRQOL, side-effects, complications and satisfaction with allocated treatment. Hysterectomy requires hospitalization of 2 to 4 days, depending on the approach [31]. On the other hand, the source of adenomyosis is removed and might provide a more definite solution. An embolization is less invasive and in general, requires hospitalization of only 1 night [31]. Meanwhile, the uterus is preserved and complaints may continue (possibly to a lesser extent). How these pros and cons relate between the two strategies is unknown. If embolization proves to be comparable in terms of HRQOL, this can be offered to patients as a less invasive alternative, in particular in women that would like to preserve the uterus. These study outcomes could inform future patients about the expected effect of UAE and hysterectomy in the treatment of symptomatic adenomyosis and could therefore support shared decision making. These results are expected to improve patients’ wellbeing and quality of life.

## Appendix

Multimedia Appendix 1 Text and animations on the website.

## Abbreviations

AMH: Anti-Mullerian hormone
eCRF: electronic clinical report forms
EMMY: Embolization versus Hysterectomy
HRQOL: Health-Related Quality of Life
MRI: magnetic resonsnce imaging
PMMA: polymethylmethacrylate
QUESTA: Quality of Life after Embolization vs Hysterectomy in Adenomyosis
SF-12: Short Form-12
TLH: total laparoscopic hysterectomy
TVUS: transvaginal ultrasound
UAE: uterine artery embolization
VH: vaginal hysterectomy
WHOQOL-bref: World Health Organization Quality Of Life-Bref
