# Supplementary figures and images for: Uterine Artery Embolization Versus Hysterectomy in the Treatment of Symptomatic Adenomyosis: Protocol for the Randomized QUESTA Trial
Source: JMIR Res Protoc. 2018 Mar 1;7(3):e47. doi: 10.2196/resprot.8512 (PMC5856934; doi:10.2196/resprot.8512)

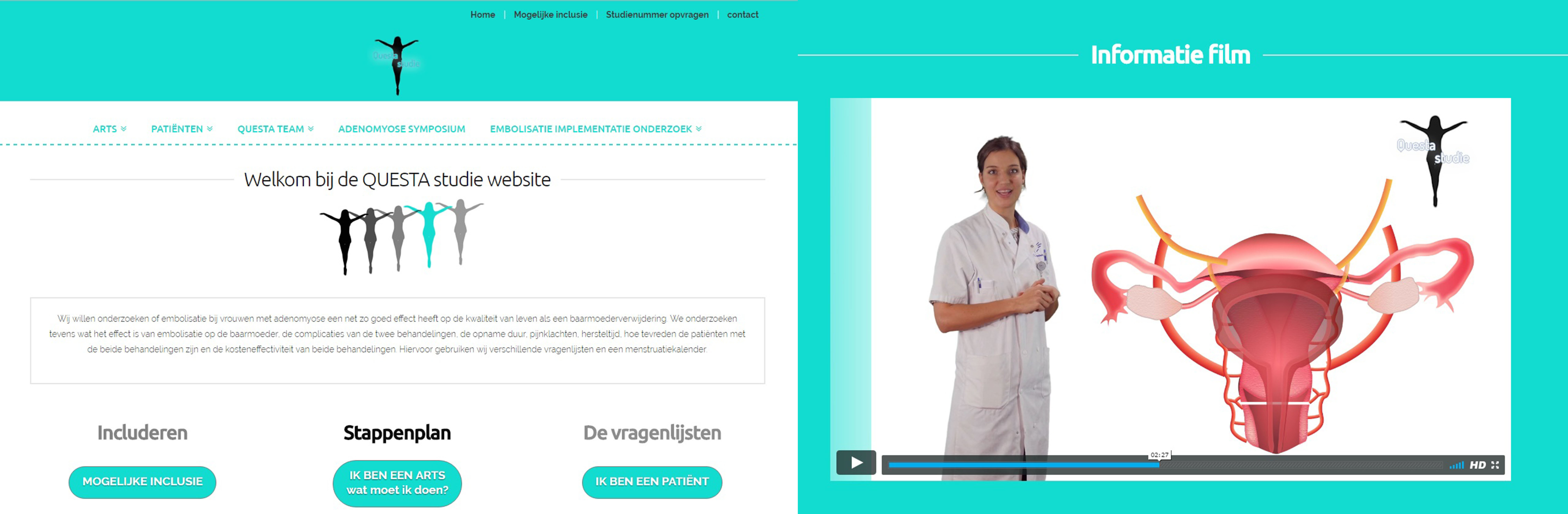

Supplement: Multimedia Appendix 1 [file resprot_v7i3e47_app1.jpg]
